# Advancements in Chitosan–Anthocyanin Composite Films: Sustainable Food Preservation with Biodegradable Packaging

**DOI:** 10.3390/foods14101721

**Published:** 2025-05-13

**Authors:** Le Li, Quanhong Li

**Affiliations:** 1 School of Environmental and Quality Testing, Chongqing Chemical Industry Vocational College, No. 2009, East Bodhi Road, Changshou District, Chongqing 401228, China; 2 College of Food Science & Nutritional Engineering, China Agricultural University, No. 17 Tsinghua Dong Road, Beijing 100083, China

**Keywords:** biodegradable films, chitosan–anthocyanin composite films, food preservation

## Abstract

To mitigate the escalating environmental pollution caused by plastic packaging films and the associated health risks of the migration of microplastics into food, the development of biodegradable food packaging materials has been recognized as an urgent research priority. In this review, recent advancements in chitosan–anthocyanin composite films (C–As) over the past decade are systematically summarized. First, the key antibacterial and antioxidant mechanisms of chitosan and anthocyanins that contribute to their functional properties are elucidated. Next, the influence of anthocyanin incorporation on the physicochemical characteristics of C–As, including mechanical strength, barrier properties, and thermal stability, is examined. Furthermore, the controlled release behavior of anthocyanins within these C–As and their implications for prolonged bioactivity are explored. Finally, the practical applications of these films in preserving fresh food, such as fruits, vegetables, and meat, are discussed. This review provides a comprehensive framework for designing and optimizing chitosan–anthocyanin-based packaging materials, offering valuable insights for developing sustainable, high-performance food preservation strategies with significant industrial and environmental implications.

## 1. Introduction

The preservation of fresh food presents significant challenges due to its complex composition, which includes moisture, nutrients, and enzymes. Food packaging plays a crucial role in maintaining the sensory attributes (e.g., appearance and texture) and nutritional quality of food. Acting as a physical barrier, packaging helps prevent oxygen, moisture, and microbial penetration, thereby delaying spoilage, preserving flavor, and extending shelf life [1,2]. However, for decades, the global food packaging industry has relied primarily on non-degradable or non-recyclable plastics such as polyethylene, polypropylene, polyvinyl chloride, and polystyrene [2]. These materials not only persist in the environment but also pose food safety risks due to plastic migration. Recent studies have detected significant concentrations of microplastics in human blood and tissues, raising serious health concerns [3]. In addition, plastics also exert significant environmental impacts, such as plastic accumulation, ecological toxicity, and persistence in ecosystems [3]. Consequently, the development of biodegradable active packaging films as sustainable alternatives to conventional plastics has become a major research focus.

Among the most promising biodegradable materials, biopolymeric polysaccharides, such as alginates, carrageenan, cellulose, chitosan, pectin, and starch, have gained attention due to their biodegradability, environmental compatibility, and safety [2]. Chitosan, a naturally derived bioactive polymer, has emerged as a particularly attractive candidate for food packaging applications. It possesses multiple advantages, including excellent biocompatibility, film-forming ability, and moisture-absorption capacity [4]. Additionally, its thickening, gelling, and antimicrobial properties further expand its functional potential. Studies have shown that chitosan effectively inhibits the growth of bacteria, fungi, molds, and yeasts by disrupting microbial cell membranes and suppressing metabolic activity, thereby extending the shelf life of food [5]. However, despite its bioactivity, chitosan exhibits relatively weaker antimicrobial effects compared to synthetic agents and has limited thermal stability and mechanical strength. To address these challenges, researchers have incorporated functional components such as essential oils, polyphenols, and nanoparticles into chitosan films, significantly enhancing their antimicrobial efficacy and mechanical performance [6]. Additionally, the inclusion of plasticizers (e.g., water, ethylene glycol, glycerol, and sorbitol) has improved the thermal stability, flexibility, and tensile properties of chitosan films, further supporting their practical applications in food packaging [2].

Anthocyanins, a class of natural phenolic compounds, have garnered increasing interest due to their potent antioxidant, anti-inflammatory, antimicrobial, and anticancer properties. Their high commercial value extends beyond food preservation to pharmaceuticals, cosmetics, and textiles [7]. In light of growing concerns over the potential risks of synthetic preservatives, anthocyanins have been explored as promising natural alternatives. Studies have demonstrated that anthocyanins not only enhance the physical and mechanical properties of chitosan–anthocyanins composite films (C–As) but also improve their antimicrobial and antioxidant capacities through controlled-release mechanisms [8,9]. Notably, C–As have shown encouraging results in fresh food preservation applications.

Despite the growing research interest in C–As, a comprehensive review of the effects of anthocyanins on their physicochemical properties remains lacking. This review aims to systematically summarize recent advances in the development of C–As, with a particular focus on the impact of anthocyanin incorporation on film properties. Additionally, it examines the controlled-release characteristics of anthocyanins and their potential applications in food preservation, providing a theoretical foundation and technical guidance for the future development of sustainable and high-performance food packaging materials.

## 2. Properties of Chitosan and Anthocyanins

### 2.1. Physical, Chemical, and Biological Properties of Chitosan

Chitosan is a naturally occurring polysaccharide polymer composed of D-glucosamine and N-acetylglucosamine units linked by β-1,4-glycosidic bonds [10]. Its chemical structure confers excellent film-forming ability, ion adsorption capacity, and moderate water solubility, which varies with molecular weight and the degree of deacetylation (DD). Higher DD chitosan (>75%) exhibits increased cationic charge density, making it more soluble in acidic conditions (pH < 6.5) and enhancing its applicability in film-forming processes [11]. Conversely, lower DD chitosan has reduced solubility but improved structural integrity, making it more suitable for composite formulations.

**Figure 1:**
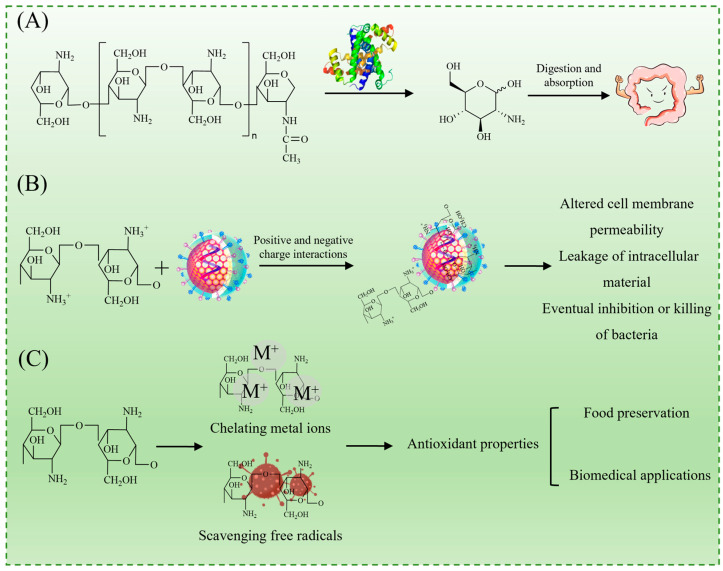
Properties of chitosan: biodegradability (**A**); antibacterial activity (**B**); and antioxidant activity (**C**).

As illustrated in Figure 1A, chitosan undergoes enzymatic degradation in natural environments and biological systems, primarily via a lysozyme, yielding small-molecule oligosaccharides that are further metabolized into non-toxic D-glucosamine [12]. This biodegradability enhances its sustainability and aligns with the growing demand for environmentally friendly materials in food and biomedical applications.

The antimicrobial activity of chitosan is primarily attributed to the protonation of its amino groups under acidic conditions, resulting in positively charged ammonium ions (–NH_3_^+^), which facilitate electrostatic interactions with the negatively charged components of bacterial cell membranes. This interaction disrupts membrane integrity, leading to increased permeability, the leakage of intracellular substances, and eventual cell death (Figure 1B) [5,13]. Additionally, chitosan has been reported to induce oxidative stress and interfere with microbial metabolic pathways, further contributing to its antimicrobial effects. It exhibits broad-spectrum activity against both Gram-positive bacteria (e.g., *Staphylococcus aureus*) and Gram-negative bacteria (e.g., *Escherichia coli*), making it highly effective against foodborne spoilage and pathogenic bacteria [5].

Beyond its antimicrobial effects, chitosan and its derivatives also display strong antioxidant properties, primarily through metal ion chelation and free radical scavenging mechanisms (Figure 1C). These properties help mitigate oxidative degradation in food products, reinforcing their potential in food preservation applications [14].

Chitosan-based films are widely utilized in food packaging due to their biocompatibility, antimicrobial properties, and moderate gas barrier performance. These films effectively extend the shelf life of perishable products such as fruits, meat, and dairy items by reducing microbial growth and oxidative damage [15]. However, their mechanical strength and water vapor barrier properties often require enhancement through blending with other biopolymers (e.g., gelatin, starch, and polyvinyl alcohol) or the incorporation of natural antioxidants. For instance, chitosan–anthocyanin composite films not only exhibit improved tensile strength and flexibility but also enhanced antimicrobial and antioxidant efficacy, further expanding their potential in food packaging applications [16].

### 2.2. Physical, Chemical, and Biological Properties of Anthocyanins

**Figure 2:**
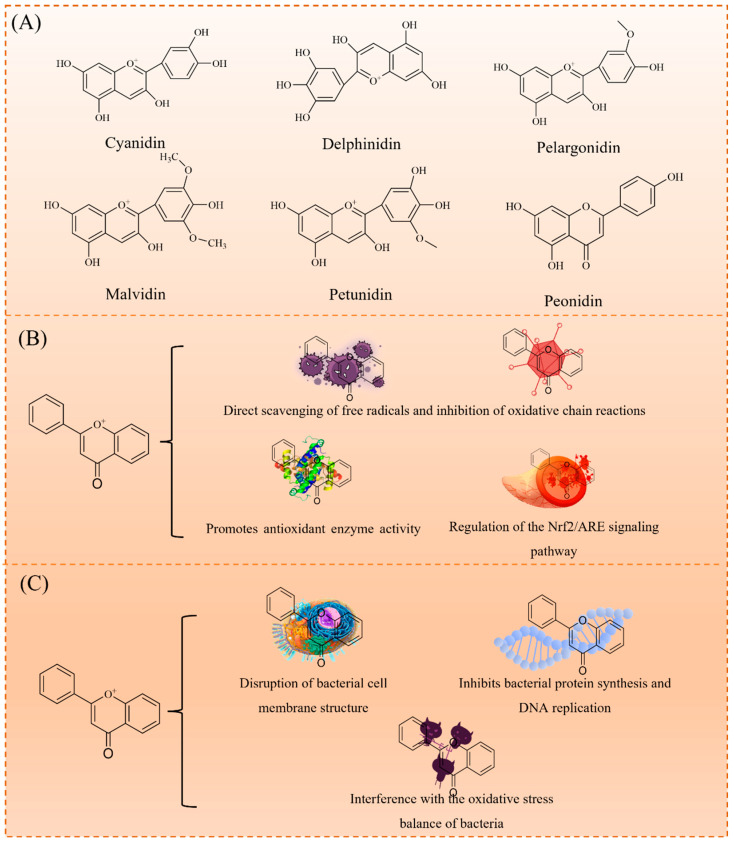
Properties of anthocyanins: classification of anthocyanins (**A**); antibacterial activity mechanism (**B**); and antioxidant mechanism (**C**).

Anthocyanins, an important subclass of flavonoids, are characterized by a 2-phenylbenzopyran (C6-C3-C6) structure, with anthocyanidin as the core. As shown in Figure 2A, they are classified into cyanidin, delphinidin, pelargonidin, malvidin, petunidin, and peonidin based on hydroxyl and methoxy substitutions. Typically, anthocyanins exist as glycosides, with a sugar moiety attached at the C3 position, enhancing their water solubility and stability. These compounds exhibit diverse biological activities, including antioxidant, antimicrobial, anti-inflammatory, anticancer, and protective cardiovascular effects [17]. These activities are largely associated with their redox properties, particularly the interconversion between quinone and hydroquinone structures, which play a key role in electron transfer. This mechanism underlies their ability to scavenge free radicals, inhibit lipid peroxidation, and modulate cellular signaling pathways, thereby contributing to their observed health benefits [18,19].

Anthocyanins are potent natural antioxidants. As illustrated in Figure 2B, their antioxidant mechanisms include (1) the direct scavenging of free radicals (e.g., superoxide anion and hydroxyl radicals) to inhibit oxidative chain reactions; (2) enhancing the activity of antioxidant enzymes (e.g., superoxide dismutase SOD, catalase CAT, and glutathione peroxidase GPx); and (3) upregulating the Nrf2/ARE signaling pathway, a key regulator of cellular antioxidant defenses. These mechanisms enable anthocyanins to effectively reduce oxidative stress and prevent chronic diseases, such as cardiovascular diseases, neurodegenerative disorders, and diabetes [18,20].

In addition to their antioxidant properties, anthocyanins exhibit broad-spectrum antimicrobial activity, effectively inhibiting both Gram-positive (e.g., *Staphylococcus aureus*) and Gram-negative bacteria (e.g., *Escherichia coli*). As shown in Figure 2C, their antimicrobial mechanisms include: (1) disrupting bacterial cell membranes, leading to cytoplasmic leakage; (2) inhibiting protein synthesis and DNA replication; and (3) interfering with bacterial oxidative stress balance. These properties make anthocyanins promising candidates for applications in food preservation, medical materials, and biomedicine.

Numerous studies have linked anthocyanin intake to various health benefits. For instance, they can lower blood pressure, improve vascular endothelial function, and reduce the risk of atherosclerosis [21]. They also inhibit cancer cell proliferation by regulating apoptosis-related proteins (e.g., Bax and Bcl-2) and blocking signaling pathways such as PI3K/Akt and MAPK [22]. Furthermore, anthocyanins reduce the deposition of β-amyloid, improving cognitive function in Alzheimer’s patients [23], and ameliorate insulin resistance and glucose metabolism, aiding in type 2 diabetes management [24]. Interestingly, anthocyanins from different sources exhibit varying performances in these studies.

In recent years, anthocyanins have been widely applied in food packaging, drug delivery, and biomedical materials. They are often combined with polymers (e.g., chitosan, gelatin, and hydroxypropyl methylcellulose) to form functional films. These functional films exhibit unique advantages in food preservation, controlled drug release, and tissue engineering due to their excellent bioactivities and biodegradability. For example, C–As not only display improved mechanical strength and barrier properties but also enhanced antimicrobial and antioxidant activities, demonstrating potential in food packaging applications [21].

## 3. Effects of Anthocyanins on the Properties of C–As

**Figure 3:**
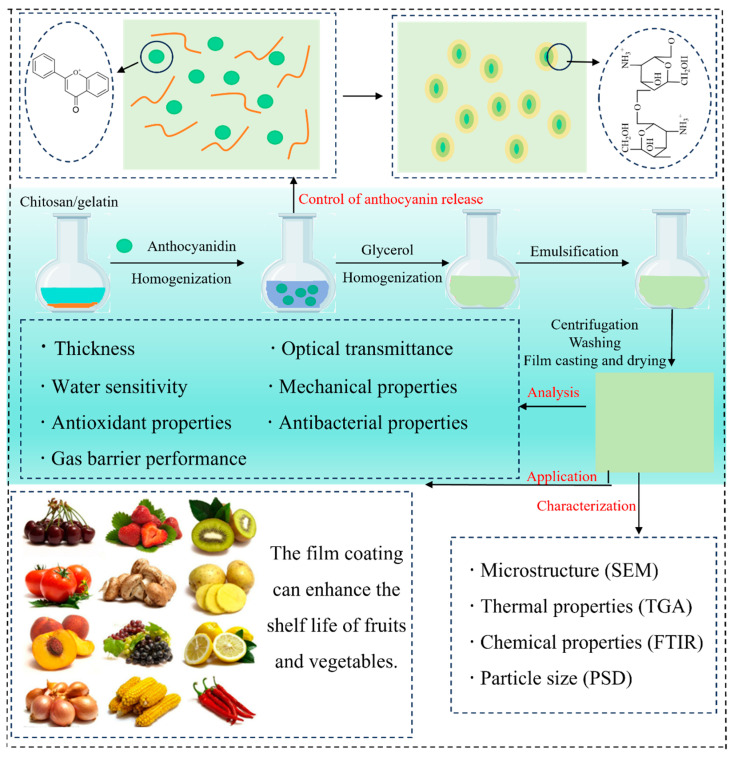
Preparation, characterization, controlled release behavior, and potential applications of C–As.

A substantial body of research has demonstrated the beneficial effects of incorporating anthocyanins into C–As. This section provides a concise summary of these enhancements, supported by the recent findings compiled in Table 1. Figure 3 illustrates the preparation process of chitosan/gelatin-based films enriched with anthocyanins, highlighting key steps and interactions.

### 3.1. Thickness

The thickness of C–As is a critical parameter influencing their mechanical properties and barrier functionality. Studies indicate that anthocyanin incorporation increases film thickness, primarily due to enhanced intermolecular interactions and structural rearrangements. Specifically, compared to pure chitosan films, the addition of anthocyanins promotes the alignment and compact binding of chitosan molecules, attributed to strengthened hydrogen bonding and hydrophobic interactions [41].

The effect of anthocyanin concentration on film thickness has been extensively studied [25,26]. For instance, increasing the anthocyanin concentration leads to the progressive thickening of the films, likely influenced by the electrostatic interactions, crosslinking frequency, and swelling effects of the film matrix [25,26,42]. A progressive increase in film thickness was observed with increasing anthocyanin concentrations, indicating a dose-dependent effect [27], a trend that is consistent with other polyphenolic composite systems.

However, under conditions of low pH or high ionic strength, anthocyanin incorporation may reduce film thickness. This phenomenon is linked to the decreased molecular stability of anthocyanins in acidic environments, enhanced aggregation effects, and weakened interactions with chitosan, ultimately affecting film uniformity and the film-forming process [43,44]. Overall, the thickness of C–A films is influenced by several factors, including the source of anthocyanins, their molecular structure, pH, and the degree of crosslinking, as summarized in Table 1. In particular, the anthocyanin source can affect film thickness due to differences in composition, purity, and the presence of co-pigments or other interacting compounds.

### 3.2. Optical Transmittance

**Figure 4:**
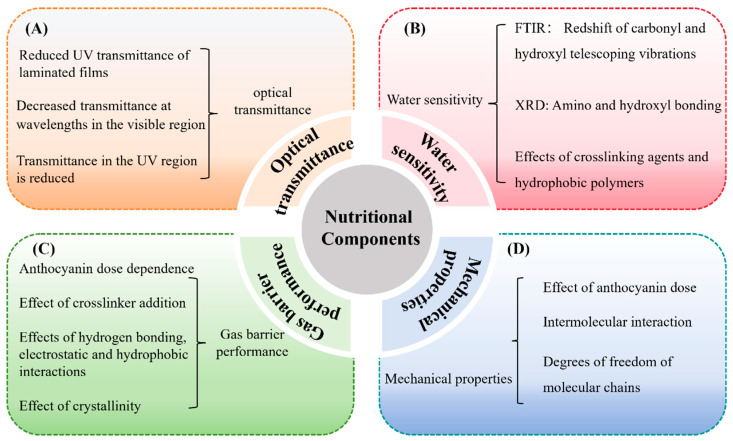
Effects of anthocyanins on the properties of C–As: optical transmittance (**A**); water sensitivity (**B**); gas barrier properties (**C**); and mechanical properties (**D**).

The UV-blocking capacity of C–As can be evaluated by measuring their optical transmittance in the 200–800 nm wavelength range. This assessment reveals both UV shielding effects and transparency in the visible spectrum. High visible light transmittance is desirable for maintaining the natural appearance of packaged food, while low UV transmittance helps mitigate UV-induced lipid oxidation, extending shelf life (Figure 4A).

UV–vis spectroscopy shows that anthocyanins reduce UV transmittance via selective absorption. For example, increasing the anthocyanin concentration from 2% to 6% (*w*/*w*) significantly reduces transmittance in the 280–400 nm range, from over 50% to below 10%, while visible light transmittance declines from over 90% to approximately 30% [28]. The antioxidant activity of anthocyanins further enhances UV-blocking efficacy by mitigating UV-induced degradation of the film structure.

C–As enriched with cyanidin, delphinidin, and mulberry anthocyanins exhibit superior UV shielding and reduced transmittance, resulting in increased opacity [29,45]. These findings consistently demonstrate that anthocyanin incorporation enhances UV-blocking properties while reducing visible light transmittance, as detailed in Table 1.

### 3.3. Water Sensitivity

Improving the water resistance of C–As has become a focal point in edible packaging development. Due to the hydrophilic nature of chitosan, its stability in high-humidity environments is limited, compromising barrier properties and mechanical strength [46]. Anthocyanin incorporation has emerged as an effective strategy to enhance water resistance (Table 1).

Studies show that unmodified chitosan films exhibit high water solubility, but the addition of 0.5–1.5 mg/mL of anthocyanins reduces solubility to below 20% [30]. As illustrated in Figure 4B, this reduction is attributed to hydrogen bonding and hydrophobic interactions between anthocyanins and chitosan, increasing the crosslinking density and limiting water penetration [47]. FTIR and XRD analyses reveal that anthocyanins interact with chitosan’s amino (-NH_2_) and hydroxyl (-OH) groups, enhancing crystallinity and water stability [31,47].

The water resistance of C–As is further improved by crosslinkers and hydrophobic polymers. For example, co-doping with gelatin reduces solubility by forming a denser network structure [48]. Additionally, incorporating natural antioxidants increases the crosslink density, further reducing solubility [49]. Compared to unmodified chitosan films, anthocyanin–chitosan composites exhibit improved water resistance, highlighting the role of anthocyanins in strengthening hydrogen bonding and hydrophobic interactions [50].

### 3.4. Gas Barrier Properties

C–As, as edible food packaging materials, effectively block oxygen, moisture, ultraviolet radiation, and microorganisms, thereby extending the shelf life of food products. Their gas barrier properties are primarily reflected in the regulation of water vapor permeability (WVP) and oxygen permeability (OP), enabling adaptation to different food storage requirements. For low-moisture foods (e.g., fresh fruits, vegetables, and baked goods), the composite film helps maintain humidity, prevent moisture loss, and preserve food quality [2]. Conversely, for high-moisture foods (e.g., meat, seafood, and dairy products), the film effectively prevents environmental moisture infiltration, reducing the risk of microbial proliferation [2]. Although vacuum packaging is widely used in food preservation, optimal packaging techniques must be refined due to variations in storage conditions among different food products.

As illustrated in Figure 4C, anthocyanin incorporation reduces WVP and OP, enhancing the gas barrier properties of chitosan films. For example, integrating anthocyanins into the chitosan matrix decreases WVP in a dose-dependent manner, with more pronounced effects under high relative humidity conditions [32]. Similarly, in crosslinked C–As, anthocyanins rich in acyl or benzene ring structures further reduce WVP by strengthening intermolecular hydrogen bonding and hydrophobic interactions [51]. Furthermore, anthocyanin concentrations exhibit a similar effect on OP. In crosslinked C–As, anthocyanin incorporation reduces OP in a dose-dependent manner [51]. This phenomenon may result from structural modifications in the chitosan molecular network induced by the addition of anthocyanins, enhancing hydrogen bonding, electrostatic interactions, and hydrophobic interactions, leading to a denser microstructure and improved barrier performance [31].

The gas barrier properties of films are closely related to their degree of crosslinking and molecular packing density, typically quantified via WVP and OP measurements. Studies have demonstrated that the addition of anthocyanins increases the crystallinity of chitosan films, thereby improving their gas barrier performance [33]. Differential scanning calorimetry analyses further indicate that anthocyanin incorporation promotes the crystalline structure of the films, reducing gas diffusion channels and enhancing gas impermeability [52].

### 3.5. Mechanical Properties

Tensile strength (TS) and elongation at break (EB) are key parameters for evaluating the mechanical performance of C–As, reflecting their strength and flexibility, respectively. TS is primarily determined by the strength of intermolecular interactions within the film matrix, whereas EB is influenced by polymer chain mobility and plastic deformation capacity. In C–A systems, strong intermolecular forces such as hydrogen bonding and electrostatic interactions typically enhance TS, while increased chain mobility and reduced crosslinking may lead to a higher EB [53].

As summarized in Table 1 and Figure 4D, several studies have reported that anthocyanin incorporation generally improves the mechanical properties of chitosan-based films. For example, Zheng et al. (2024) [33] observed an increase in TS and a decrease in EB with a higher anthocyanin content, attributing this to enhanced hydrogen bonding that improves structural integrity, while the anthocyanins’ plasticizing effect partially offsets rigidity. Conversely, other studies have reported simultaneous increases in both TS and EB, such as in films incorporating blueberry anthocyanins [54] or blood orange anthocyanins [53], suggesting that the impact of anthocyanins may vary depending on their source and chemical composition.

However, inconsistencies among findings indicate that the effect of anthocyanins on mechanical performance is not universally predictable. Variability in extraction methods, anthocyanin purity, degree of polymerization, and incorporation techniques (e.g., direct mixing vs. nanoencapsulation) may significantly influence film structure and interactions. For instance, the addition of excessive amounts of anthocyanins has been shown to disrupt the native chitosan network, leading to weakened intermolecular interactions and simultaneous reductions in both TS and EB [50,55].

Therefore, while many studies suggest that anthocyanin incorporation can enhance the mechanical properties of C–A films, it is essential to consider formulation-specific variables and methodological differences when interpreting these results. A more systematic evaluation of anthocyanin concentration, source, and processing conditions is needed to clarify these effects and ensure reproducibility for food packaging applications.

### 3.6. Thermal Properties

The addition of anthocyanins significantly enhances the thermal stability of C–As. As shown in Table 1, increasing the anthocyanin content raises the primary thermal degradation temperature (Tmax) of C–As by 10–30 °C compared to pure chitosan films, indicating a substantial improvement in thermal performance [54]. The thermogravimetric analysis further reveals that anthocyanin incorporation delays the dehydration process of C–As and increases the onset decomposition temperature in the first thermal degradation stage [54]. These findings suggest that anthocyanins stabilize the film structure through intermolecular hydrogen bonding and hydrophobic interactions at low temperatures and may provide free radical scavenging capacity at high temperatures, thereby inhibiting thermo-oxidative degradation.

As indicated in Table 1, the presence of anthocyanins does not compromise the thermal stability of chitosan films. For example, Xie et al. (2023) [54] found that cabbage anthocyanins interact with the chitosan matrix to enhance Tmax. Additionally, Tavassoli et al. (2024) [32] reported that anthocyanin-containing chitosan–carboxymethyl cellulose C–As exhibited superior thermal stability. Similarly, synergistic interactions between anthocyanins and other components (e.g., polyphenols and nanoparticles) further enhanced the film’s thermal resistance [56]. For instance, the thermal shielding effect of zinc oxide nanoparticles and the structural reinforcement provided by nanocellulose improved the film’s thermal degradation behavior [57].

Under high-temperature conditions, the polyphenolic structure of anthocyanins may mitigate the thermo-oxidative degradation of the film material via free radical scavenging [58]. This effect is observed in various C–As, although the degree of enhancement depends on the anthocyanin’s concentration, molecular structure, and interaction with the chitosan matrix. Therefore, optimizing anthocyanin loading is crucial for developing food packaging materials with high thermal stability.

### 3.7. Microstructural Characteristics

Anthocyanin incorporation significantly alters the microstructure of C–As. Scanning electron microscopy and atomic force microscopy analyses indicate that anthocyanin integration results in smoother, denser film surfaces with reduced surface roughness [59]. This phenomenon is likely due to hydrogen bonding and electrostatic interactions between anthocyanins and chitosan molecules, enhancing the compactness of the film matrix [60].

## 4. Controlled Release of Anthocyanins and Their Potential Applications

Anthocyanins hold significant promise in food packaging and pharmaceutical applications. However, their photosensitivity and thermal instability limit their practical use. Incorporating anthocyanins into chitosan C–As has emerged as an effective stabilization strategy, leveraging the synergistic properties of both components.

The structure of anthocyanins (e.g., hydroxyl and carbonyl groups) endows them with excellent antioxidant properties. However, anthocyanins from different sources exhibit varying performances in C–As. For example, Li et al. (2024) [61] found that blueberry anthocyanins demonstrated higher antioxidant capacity than black goji anthocyanins, likely due to differences in the number and arrangement of hydroxyl groups. Similarly, Deng et al. (2024) [62] observed that chitosan films loaded with anthocyanins effectively inhibited microbial growth, enhancing the functional properties of the films.

In recent years, increasing attention has been paid to the release behavior of anthocyanins, which plays a crucial role in their bioavailability and functionality. To address challenges related to stability and controlled release, nanoparticles have been extensively applied as effective delivery systems for anthocyanins. These nanocarriers not only reduce direct interactions between anthocyanins and chitosan but also modulate anthocyanin release kinetics by influencing film porosity and hydration behavior (Figure 3). For instance, Zhang et al. (2023) [63] reported that encapsulating anthocyanins in nanoparticles significantly prolonged their release duration, extending it by 10 h compared to conventional blended films. Additionally, Atay et al. (2018) [9] found that nano-composite films prepared using a chitosan–gelatin matrix effectively reduced the release rate of blueberry anthocyanins.

Overall, C–As integrate the bioactivity of anthocyanins with the film-forming properties of chitosan, demonstrating broad application potential in food packaging and biomedical materials. Future research should further refine anthocyanin release control strategies by incorporating different types of nanocarriers, crosslinking agents, or stimuli-responsive systems to enhance the stability and functionality of composite films.

## 5. Applications of Anthocyanin-Containing C–As in Fresh Food Preservation

C–As have been widely utilized in the food packaging industry. For example, many food companies use chitosan films containing blueberry anthocyanins to package fruits, vegetables, meat, and dairy products [19,64,65]. Additionally, chitosan films incorporated with black goji anthocyanins have been extensively applied to preserve fresh agricultural products [66].

**Figure 5:**
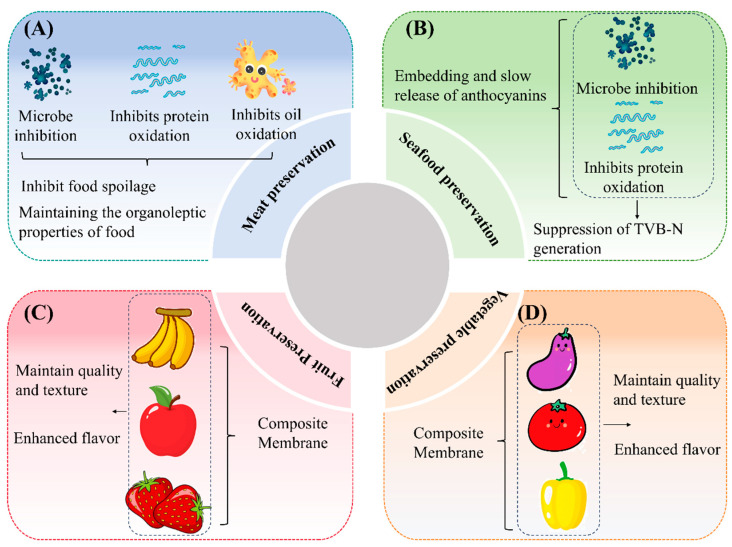
Applications of anthocyanin-containing composite films in fresh food preservation: meat preservation (**A**); seafood preservation (**B**); fruit preservation (**C**); and vegetable preservation (**D**).

In recent years, extending the shelf life of fresh food has become a key focus in the food industry. The development of C–As with preservation capabilities is particularly important due to their potential applications. Studies have shown that these films exhibit outstanding performance in preserving various fresh foods, including fruits, vegetables, meat, and seafood [2,26]. For instance, fresh meat is highly susceptible to microbial contamination, leading to protein and lipid oxidation, which accelerates spoilage and results in resource waste. Comparative studies have demonstrated that C–As containing black goji or purple tomato anthocyanins effectively inhibit microbial growth in pork [67]. Similarly, films containing black rice bran anthocyanins significantly suppressed microbial proliferation in pork [68]. These findings indicate that anthocyanin-rich C–As can effectively inhibit microbial growth, thereby prolonging the shelf life of food (Figure 5A). Notably, anthocyanin incorporation also helps maintain the key sensory attributes of food, such as flavor, color, texture, and juiciness [68].

Moreover, C–As exhibit excellent performance in seafood preservation (Figure 5B). For example, when storing fresh *Pelteobagrus fulvidraco* at 4 °C for nine days, fish fillets wrapped in standard chitosan films showed a significant increase in total volatile basic nitrogen (TVB-N) levels, whereas those packaged with anthocyanin-infused chitosan films maintained lower TVB-N levels [69]. This effect is attributed to the gradual release of anthocyanins from the composite film. Compared to conventional blended films, nanocarrier-encapsulated anthocyanin–chitosan films enable the controlled release of anthocyanins, thereby enhancing their sustained release properties while effectively preserving food quality and delaying spoilage.

The preservation efficacy of C–As can be attributed to multiple factors: (I) the antioxidant activity of anthocyanins, (II) their antimicrobial properties, (III) the film-forming and barrier properties of chitosan, (IV) the synergistic effects between anthocyanins and chitosan, (V) controlled release mechanisms, and (VI) the direct inhibition of microbial growth on food surfaces. As biopolymer-based active coatings, these C–As adhere directly to the food surface, effectively extending the shelf life of fruits and vegetables by inhibiting microbial proliferation and slowing oxidation reactions.

As illustrated in Figure 5C,D, C–As show great potential in maintaining fruit and vegetable quality and prolonging storage life. For instance, compared to bananas wrapped in pure chitosan films, those packaged with C–As exhibited superior color retention and firmness, along with a higher polyphenol content [70]. Similar effects were observed in strawberries, where the composite film not only improved the fruit’s appearance and texture but also enhanced flavor in sensory evaluations [71]. Additionally, the incorporation of black goji anthocyanins into C–As effectively suppressed microbial growth, improved fruit quality, and enhanced antioxidant activity, further reducing decay [41].

In summary, C–As exhibit strong potential for food packaging applications due to their demonstrated ability to prolong shelf life, preserve food quality, and provide antioxidant protection. However, their translation from laboratory development to commercial use remains limited. Key challenges include achieving consistent large-scale film fabrication with uniform anthocyanin distribution, ensuring regulatory compliance related to bioactive compound migration and food contact safety, and validating performance under industrial processing and real-world storage conditions. Addressing these limitations is essential to unlocking the full commercial potential of C–As in modern food packaging systems.

## 6. Conclusions

This review underscores the multifaceted role of anthocyanins in enhancing chitosan-based active films (C–As), contributing to improved optical, mechanical, barrier, and bioactive properties. The performance of these films is intricately linked to the source and molecular structure of anthocyanins, their spatial distribution, and their interactions with the chitosan matrix and other co-components. A key consideration is the optimization of anthocyanin concentration to balance bioactivity with film integrity, which varies across formulations and extraction methods.

While antioxidant and antimicrobial properties are central to anthocyanin function, recent research suggests that their contributions extend beyond direct bioactivity, mediating intermolecular interactions, controlling release kinetics, and enhancing structural stability under storage and usage conditions. However, reported effects remain inconsistent due to variability in anthocyanin profiles, incorporation techniques, and test protocols, indicating a need for standardized characterization frameworks and deeper mechanistic understanding.

Despite their promise, the practical deployment of C–As in food packaging faces several unresolved challenges. Technically, anthocyanins are inherently unstable under fluctuating pH, thermal, oxidative, and photolytic environments, leading to the loss of function. Their partial miscibility with chitosan can result in heterogeneous matrices, compromising film performance. Economically, large-scale production is hindered by low anthocyanin extraction yields, seasonal sourcing constraints, and a lack of scalable encapsulation or coating systems. Furthermore, regulatory pathways for edible films containing plant-derived bioactives remain underdeveloped in many jurisdictions, particularly regarding compound migration, safety validation, and labeling.

To advance the field, future research must address these multi-level bottlenecks. Technologically, integrating micro- and nanoencapsulation strategies with intelligent stimuli-responsive systems could stabilize anthocyanins and enable dynamic food monitoring. Material innovations should explore compatibilizers or co-polymer systems that improve molecular dispersion and mechanical uniformity. From a translational perspective, pilot-scale validations, lifecycle assessments, and alignment with international food packaging regulations (e.g., EFSA and FDA) are essential to move C–As from laboratory innovation to commercial application. A cross-disciplinary roadmap linking materials science, food technology, and regulatory science will be critical to unlocking the full potential of anthocyanin-functionalized packaging materials.

## Figures and Tables

**Table 1:** Recent studies on the effect of anthocyanin on the performance of C–As.

Type of Polymer	Type of Anthocyanin	Dose of Anthocyanin	Effects on CEOs	Reference
Chitosan	Purple eggplant anthocyanin	1%, 3%, 5% (*w*/*w*)	Thickness (↑), UV (↓), WVP (↑), TS (↑), EB (↓), Thermal properties (↓)	[25]
Chitosan	Black eggplant anthocyanin	1%, 3%, 5% (*w*/*w*)	Thickness (↑), UV (↓), WVP (↑), TS (↑), EB (↓), Thermal properties (↓)	[25]
Chitosan/Gelatin	Blueberry anthocyanin	5%, 10%, 15% (*w*/*w*)	Thickness (↑), UV (↑), WVP (↑), TS (↑), EB (↓), Thermal properties (↓)	[26]
Chitosan/Polyvinyl Alcohol	Black carrot anthocyanins	-	TS (↓), Thermal properties (↑)	[27]
Chitosan	*Hibiscus sabdariffa* L.anthocyanin	-	UV (↓)	[28]
Chitosan/hydroxyethyl cellulose/titanium dioxide nanoparticles	Mulberry anthocyanins		UV (↓)	[29]
Starch/Chitosan	Black wolfberry anthocyanin		UV (↓), WVP (↑), TS (↑)	[30]
Carboxymethyl chitosan/nano magnesium oxide	Red kale anthocyanins	0.2% (*w*/*w*)	UV (↓), Gas Barrier Performance (↑), Thermal properties (↑), TS (↑), EB (↓)	[30]
Chitosan/chitin nanofibers	Eggplant skin anthocyanin	0.1% (*w*/*w*)	UV (↓), Gas Barrier Performance (↑), Thermal properties (↑)	[31]
Pectin/chitosan nanofibers	lacC-Ase anthocyanin	3% (*w*/*v*)	UV (↓), Gas Barrier Performance (↓), TS (↑), EB (↑), Thermal properties (↑)	[32]
Chitosan/Cellulose Nanocrystals	-	0.2% (*w*/*w*)	Thickness (↑), UV (↓), Gas Barrier Performance (↑), Thermal properties (↑)	[33]
Chitosan	purple rice anthocyanin	1%, 3%, 5% (*w*/*w*)	Thickness (↑), UV (↓), Gas Barrier Performance (↑), WVP (↑), TS (↑), Thermal properties (↑)	[34]
Chitosan	Black Rice anthocyanin	1%, 3%, 5% (*w*/*w*)	Thickness (↑), UV (↓), Gas Barrier Performance (↑), WVP (↑), TS (↑), Thermal properties (↑)	[34]
Chitosan/Starch/Gelatin	Raspberry anthocyanin	-	Thickness (↑), UV (↑)	[35]
Chitosan/titanium dioxide nanoparticles	black plum peel anthocyanins	-	UV (↓), Gas Barrier Performance (↑), Thermal properties (↑)	[36]
Agar/Sodium Alginate Chitosan	purple yam anthocyanin	-	UV (↓), Gas Barrier Performance (↑), WVP (↑), Thermal properties (↑)	[37]
Chitosan	Purple Tomato anthocyanin		Thickness (↑), UV (↓), Gas Barrier Performance (↑), WVP (↑), TS (↑), EB (↓), Thermal properties (↑)	[38]
Chitosan/Methylcellulose	Phyllanthus reticulatus anthocyanin	-	UV (↓), WVP (↑)	[39]
Chitosan/polyvinyl alcohol	*Clitoria ternatea* L. anthocyanin		Thickness (↑), UV (↑), WVP (↑)	[40]

(UV): Ultraviolet–vis blocking capability, (WVP): water vapor permeability, (TS): tensile strength, (EB): elongation at break, (↑): increase, (↓): decrease.

## Data Availability

The original contributions presented in this study are included in the article. Further inquiries can be directed to the corresponding authors.
